# Protection of renal damage by HMG-CoA inhibitors: A comparative study between atorvastatin and rosuvastatin

**DOI:** 10.22038/IJBMS.2019.38239.9080

**Published:** 2020-02

**Authors:** Maryam Jabarpour, Nadereh Rashtchizadeh, Amir Ghorbani haghjo, Hassan Argani, Mahboub Nemati, Siavoush Dastmalchi, Leila Roshangar, Masoumeh Ranjbarzadhag, Mehran Mesgari-Abbasi, Nasrin Bargahi, Davoud Sanajou

**Affiliations:** 1 Department of Biochemistry, Faculty of Medicine, Tabriz University of Medical Sciences, Tabriz, Iran; 2 Biotechnology Research Center, Tabriz University of Medical Sciences, Tabriz, Iran; 3 Urology and Nephrology Research Center, Beheshti University of Medical Sciences, Tehran, Iran; 4 Food and Drug Safety Research Center, Tabriz University of Medical Sciences, Tabriz, Iran; 5 Stem Cell Research Center, Tabriz University of Medical Sciences. Tabriz, Iran; 6 Drug Applied Research Center, Tabriz University of Medical Sciences, Tabriz, Iran

**Keywords:** Atherogenic diet, Atorvastatin, Chronic kidney disease, Hypercholesterolemia Rosuvastatin

## Abstract

**Objective(s):**

Hypercholesterolemia is a common metabolic disorder in developing and developed countries and is associated with the increased rates of chronic kidney disease (CKD). Statin therapy could reduce cholesterol synthesis as well as progression of CKD. Diversity between statins causes variety in pharmacokinetics and pharmacodynamics and also their pleiotropic effects. In the present investigation we aimed to evaluate the protective potentials of both atorvastatin (Ator) (as lipid-soluble statin) and rosuvastatin (Ros) (as water-soluble statin) against renal histopathological damages in the high cholesterol diet induced hypercholesterolemic rats (HCDIHR).

**Materials and Methods:**

Serum lipid profile, oxidized low density lipoprotein (OX-LDL), malondialdehyde (MDA), urea and creatinine levels, as well as renal histopathology were evaluated.

**Results:**

While Ros acted better than Ator to reduce serum low density lipoprotein cholesterol (LDL-C) (*P<*0.01), atherogenic index (AI) (*P<*0.01), MDA (*P<*0.01), and OX-LDL (*P<*0.01); no significant differences were noted in their cholesterol (*P=*0.72), triglyceride (TG) (*P=*0.79), and very low density lipoprotein cholesterol lowering (VLDL-C) (*P=*0.79) and high density lipoprotein cholesterol elevating effects (HDL-C) (*P=*0.72). Ator was more effective to reduce renal histopathologic indices compared to Ros, including accumulation of lipid droplet, glomerular foam cells, mesangial cell proliferation, renal hemorrhage, and tubulointerstitial damages in the kidneys of diet induced hypercholesterolemic rats.

**Conclusion:**

The findings underline that the lipophilic Ator may performs better than Ros in attenuating renal damages in HCDIHR.

## Introduction

Hypercholesterolemia, a metabolic disorder, is one of the most prevalent diseases both in developing and in developed countries. Hypercholesterolemia could be verified by investigating the serum lipid profile as decreased levels of serum high density lipoprotein (HDL) together with elevations in serum total cholesterol (TC), low-density lipoprotein (LDL), and very low density lipoprotein (VLDL) levels denote the condition (1, 2). Hypercholesterolemia and oxidative stress play pivotal roles in the induction and development of the vascular and renal diseases; moreover, they are known to be important cardiovascular risk factor in the patients with chronic kidney diseases (CKD) (3, 4).

Reactive oxygen species (ROS) are involved in the pathogenesis of various diseases. Diverse conditions could potentially result in hypercholesterolemia including obesity, high-calorie diet, genetic disorders, lifestyle, and low physical activity (1, 2); under hypercholesterolemic states an imbalance between ROS production and anti-oxidant defense system ensues that leads to persistent low grade oxidative stress (5, 6). The breakage of polyunsaturated fatty acids due to the interaction between ROS and endothelial cell membranes alters the structure and function of the endothelial lining of the vessels (7, 8).

Furthermore, elevated levels of ROS augment the production of oxidized low-density lipoproteins (OX-LDL) inside the vessel walls. With the increased levels of OX-LDL, endothelial cells express more adhesion molecules on their cell surfaces; increased numbers of monocytes differentiate into macrophages, and the smooth muscle cells begin to proliferate. These alterations result in vascular inflammation, endothelial dysfunction and ultimately facilitate the development of atherosclerosis and cardiovascular disease (CVD) (8, 9). Furthermore, increased levels of OX-LDL in the kidneys could potentially cause glomerular sclerosis in long-term (3, 10). Therefore, thorough attempts to find a suitable drug for preventing or minimizing the vascular damages associated with lipid disorders especially hypercholesterolemia is ongoing.

Statins are used for the management of hypercholesterolemia and for patients with high levels of LDL (2). Statins act by reducing the endogenous biosynthesis of cholesterol through inhibition of the 3-hydroxy-3-methylglutaryl coenzyme A (HMG-CoA) reductase, necessary enzyme for the synthesis of cholesterol in mevalonate pathway (2). It has been revealed that statins have pleiotropic effects on different types of cells. Some of these hypo-cholesterolemic-independent effects include improvements in endothelial function, alleviation of oxidative stress and inflammation, attenuation of vascular thrombogenicity, enhancements in vasomotor performance, and suppression of the extracellular matrix production (2, 11, 12).

In the present investigation, we aimed to examine the protective potentials of the hydrophilic rosuvastatin (Ros) and lipophilic atorvastatin (Ator) on the renal histopathological damages in the diet induced hyperlipidemic male rats. In general, statins include two lipophilic and hydrophilic groups; atorvastatin, lovastatin, and simvastatin are relatively lipophilic, while pravastatin and rosuvastatin are hydrophilic (13). While all statins have a common mechanism of action, they differ in their chemical structure and hydrophilic/lipophilic properties, which leads to differences in pharmacokinetics and pharmacodynamics, as well as pleiotropic effects (14, 15). Awareness of these differences may help to select the appropriate statin in different patient pathological conditions. Among these differences is that rosuvastatin has a longer half-life of about 19 hr compared with atorvastatin (13). Rosuvastatin is metabolized with cytochrome P450 2C9 and 2C19, and its main metabolite is N-desmethyl rosuvastatin, whereas atorvastatin is metabolized to cyp3a4 and its major metabolites are 2 hydroxy, 4 hydroxy-atorvastatin acid (13, 14, 16). Hydrophilic statins in the liver are absorbed more than other tissues, which are likely to affect statin solubility profile in liver selection; in these statins, the absorption by a hydraulic carrier is the most important mechanism (13, 15, 17). In contrast, lipophilic statins enter in cells through passive diffusion (13, 17).

The renal protection properties of statin are controversial by recent observations. Studies show that statin therapy reduces the risk of developing a kidney disease and possibly statins has kidney protection effects (18, 19).

## Materials and Methods

***Experimental design and the study protocol***

Forty male Sprague-Dawley rats with an initial body weight of 160-200 were purchased from the Pastor institute, Iran. The Animal-Human Ethics Committee of Tabriz University of Medical Sciences approved the study protocol. According to Guide for care and use of Laboratory Animals [US Department of Health, Education, and Welfare, Publication number 78-23, National Institutes of Health, revised 1978] and local guidelines for compassionate use of animals in research, the animals were housed in a room maintained at standard environmental conditions in constant temperature of 22±2 ^°^C, humidity of 60-70%, and 12 hr light/dark cycles. These rats were housed in the stainless steel cages and allowed for free access to standard diet and tap water. After a week of acclimatization, all rats were weighed and their serum lipid profiles were assessed. Rats were assigned into 5 groups of 8 in each including I- control: fed with normal diet; II- sham: fed with normal diet and water gavages; III- HCD (high cholesterol diet); IV- HCD+Ros (20 mg/kg/day); V- HCD+Ator (20 mg/kg/day). Various clinical and experimental studies have shown that at equal dosages rosuvastatin is much stronger than atorvastatin in improving lipid profile. In this study, to compare the effect of lipophilic and hydrophilic statins on the protection of renal function, the same dose of 20 mg was used (20, 21). The optimal doses for Ros and Ator were adopted according to the previously published studies (20, 21).

For the induction of the hypercholesterolemia, the rats in groups III, IV, and V were fed with HCD (5% cholesterol+0.1% cholic acid) diet for 8 weeks. Hypercholesterolemia was verified by assessing the serum of lipid profiles. From the week 9, rats in groups IV and V were received Ros and Ator by intra-gastric gavages for 10 weeks, respectively.

***Blood and sample collection***

Blood samples were collected at weeks 1 and 8; the rats were fasted for a night (12 hr) with free access to water; then were anesthetized by a single IM injection of ketamine and midazolam. Blood samples were collected from the retro–orbital plexus using a micro capillary tube. Serum was separated by centrifugation at 3000 rpm for 15 min. At the ultimate day of the study, fasting blood samples were collected from the cardiac ventricles. And then, they were euthanized with the overdose of ketamine. The kidneys were dissected and washed with normal saline; the right kidneys were fixed in10% buffered formalin for histology and the left ones were frozen quickly in the liquid nitrogen to be stored at -80 for the further analyses.

***Chemistry profile and measurement of the oxidative stress parameters***

Serum levels of cholesterol, triglyceride, HDL-C, urea, creatinine (Cr), calcium (Ca), and phosphate (P) were assessed by using the commercially available kits according to the manufacturer’s instructions. VLDL-C concentrations were calculated as triglyceride (TG) concentrations/5. LDL-C was calculated using the Fridewald’s equation and the atherogenic index (AI) was measured using Schulpis and Karikas formula (22).

Serum levels of malondialdehyde (MDA) were measured by using the Lapenna method with the absorbance spectrophotometry at 535 nm (23). Additionally, serum levels of Ox–LDL were quantified with an enzyme-linked immunosorbent assay (ELISA) based method using a commercial kit (ZellBio, Ulm, Germany).

***Kidney histology***

Four to six µm tissue sections were mounted on the glass slides. Sections were stained with the hematoxylin and eosin solutions. Histological indices were blindly evaluated by an expert pathologist under light microscopy at ×400 magnification.

In order to determine tubulointerstitial damage (TID), each slide was scored semi-quantitatively from 0-3 depending on the severity of the changes including 0 without pathologic changes, 1 alteration in the field was less than 25%, 2 tissue alterations were 25-50%, and 3 renal structural alterations were 50-100%. The TID index of each sample of the kidney tissue was expressed as a mean value of all the scores.

***Statistical analyses***

All data were analyzed using the SPSS software version 16. Results were expressed as mean±SD. Comparisons between baseline serum biochemical parameters and their changes after 8 weeks of treatment were done by the Wilcoxon. Comparison between statin treatments were done by the Man-Whitney. Between groups comparisons were done by the ANOVA followed by Tukey’s *post hoc* test. *P* values of <0.05 were considered to be statistically significant.

## Results

***HCD effect on the lipid profile***

**Figure 1:**
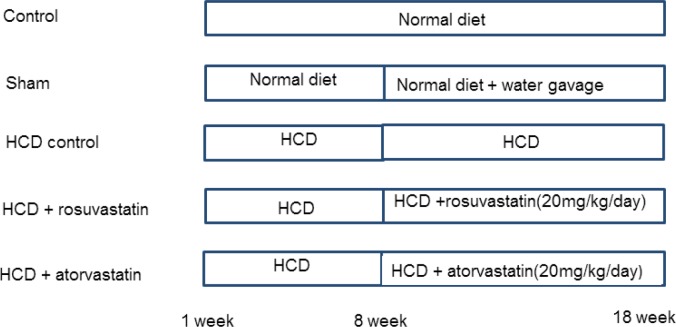
Study design

The flowchart of this study is shown in Figure 1. Fasting serum lipid profiles of the rats fed with 5% cholesterol and 0.1% cholic acid (HCD group) and rats fed with standard diet (control group) at weeks 1 and 8 of the study are shown in Table 1. Our findings revealed no differences in the serum levels of cholesterol, TG, HDL-C, LDL-C, and VLDL-C between group I (control) and group III (HCD) at week one; however, significant increases were noted in the serum cholesterol and LDL-C levels (*P*=<0.01) in group III (HCD group) compared with group I (control group); simultaneously, there was a significant reduction in the HDL-C levels (*P*<0.01) at week 8. These findings confirmed the induction of hypercholesterolemia in HCD fed animals (Table 1).

***Effects of Ros and Ator on body weight***

As shown in Table 2, significant increases in the body weight of groups III (HCD group), IV (HCD+Ros), and V (HCD+Ator group) were observed as compared to group II (sham). Treatment with the two drugs had no significant effects on the body weight of animals.

***Effects of Ros and Ator on the biochemical parameters***

A significant rise in serum levels of cholesterol, LDL-C, and AI was noted in groups III (HCD), IV (HCD+Ros), and V (HCD+Ator group) as compared to the group II (sham) (*P*<0.01). Conversely, serum levels of HDL-C had been decreased significantly in groups III (HCD), IV (HCD+Ros), and V (HCD+Ator) comparing to group II (sham) (*P*<0.01). Serum TG and VLDL-C levels showed no significant differences among the mentioned groups after HCD consumption (Table 2).

As seen in Table 2, treatment with Ros and Ator reduced serum levels of cholesterol, LDL-C, TG, VLDL-C, and AI (*P*<0.01) and significantly increased serum levels of HDL-C (*P*<0.05) in groups IV (HCD+Ros) and V (HCD+Ator) compared to group III (HCD). While Ros was more effective than Ator in reducing serum levels of LDL-C and AI (*P*<0.01), the two agents decreased serum cholesterol, TG, and VLDL-C levels and elevated serum HDL-C almost to the same degree.

There were no significant differences in serum urea, Cr, Ca, and P levels between groups III (HCD), IV (HCD+Ros), and V (HCD+Ator) compared to group II (sham) after HCD consumption (Table 3). Also no significant differences in urea, Ca, and P serum levels were seen between groups IV and V compared to group III (HCD) after treatment (Table 3). A slight albeit statistically insignificant decrease was noticed in serum Cr levels in groups IV (HCD+Ros) and V (HCD+Ator) compared to group III (HCD group). Similarly, the difference between serum Cr levels between groups IV (HCD+Ros) and V (HCD+Ator) recognized not to be statistically significant.

In order to determine the changes in oxidative stress parameters, serum MDA and OX-LDL levels were measured (Table 4). A significant increase was observed in MDA and OX-LDL levels in groups III (HCD), IV (HCD+Ros), and V (HCD+Ator) comparing to the group II (sham) after HCD consumption (*P*<0.01). After treatment, however, reductions in serum MDA and OX-LDL levels were noted in groups IV (HCD+Ros) and V (HCD+Ator) compared to group III (HCD group) (*P*<0.01). It is noteworthy that this decrease was significantly higher in the group IV (HCD+Ros) comparing to the group V (HCD+Ator) (*P*<0.01).

***Effects of Ros and Ator on renal histopathology***

**Figure 2:**
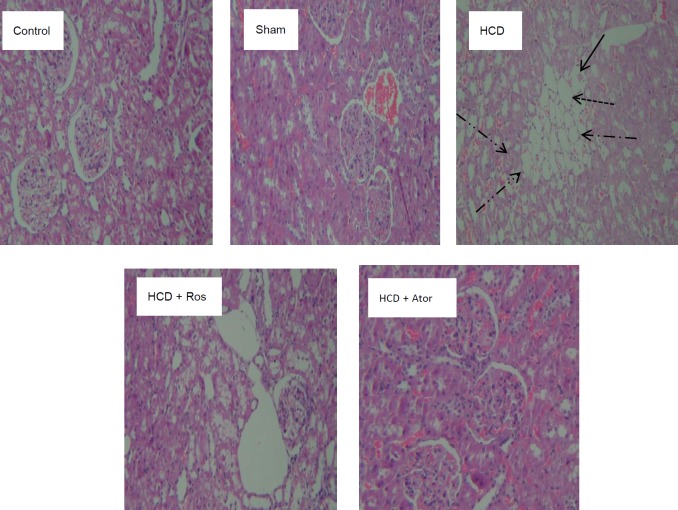
Representative histologic images (original magnification x200) H&E stained renal sections

As shown in Figure 2, normal kidney architecture was observed in control and sham animals. This study found that HCD feeding caused histopathological changes such as deposition of numerous lipid droplets especially in proximal tubules, hemorrhage, nuclear pyknosis, karyolisis, infiltration of inflammatory cells, foam cells accumulation in the glomeruli, and increased mesangial cell proliferation. Also vacuolated cytoplasm of proximal and distal tubules, tubular atrophy associated tubular dilatation and loss of brush border in tubular epithelium was seen as compared to the control and sham groups.

TID score significantly increased in group III (HCD) in comparison with groups I (control) and II (sham) (Table 5) that were alleviated by Ros and Ator. The alleviations were significantly higher in the group V (HCD+Ator) as compared to the group IV (HCD+Ros).

## Discussion

Hypercholesterolemia is a major risk factor in the cardiovascular and renal diseases. It is associated with the specific lipid profile of the metabolic disorders. In this study, feeding of male rats with HCD led to significant increases in body weight and serum levels of cholesterol and LDL-C and at the same time, it resulted in significant reductions in serum levels of HDL-C in HCD group compared to the control groups, indicating the induction of hypercholesterolemia. Meanwhile, no significant differences were observed in serum levels of TG and VLDL-C in the hypercholesterolemic rats. Moreover, HCD consumption significantly increased AI levels in HCD group comparing to the sham rats. Elevations in AI levels lead to the deposition of foam cells, plaque formation, and infiltration of lipids in the critical organs such as kidneys. With the elevations in AI levels, the probability of oxidative damage increases that leads to cardiovascular diseases (1). Other studies have shown that there is a correlation between hyperlipidemia and development of kidney diseases (10, 24). Accumulation of the lipids in the cells causes a chronic inflammation, up-regulates leptin expressions, and augments insulin resistance with the resultant dysfunctions in the kidney tubular endothelial cells (24, 25). It seems that hyperinsulinemia in rats increases blood pressure and renal sodium absorption leading to excessive increases of glomerular filtration rate. These alterations maybe responsible for the development of glomerulosclerosis (25).

This study showed that simultaneous treatment with Ros and Ator significantly reduced serum levels of cholesterol, LDL-C, and VLDL-C. These agents act by reducing cholesterol synthesis in the liver through the inhibition of the HMGCOA reductase enzyme. On the other hand, by increasing numbers of LDL receptors in liver cells, absorption rate and catabolism of LDL-C in the liver could be augmented that leads to synthesis prevention (25-27). Overall, Ros and Ator cause diminutions of lipid deposits in the kidneys (28). The diminution in the serum levels of lipid indices with Ros was significantly higher compared to Ator probably due to its sulfur content with multiple connection sites; therefore, Ros has a high affinity to be bound to the active site of the HMGCOA (29).

In this study, serum HDL-C levels increased significantly with Ros and Ator treatments compared to HCD group (*P*<0.01). It is obvious that HDL-C has crucial roles in the reverse transport of cholesterol and preventing development of atherosclerotic plaques. However, HDL-C reduces the expression of adhesion molecules in the tubular and vascular endothelial cells through its anti-oxidant and anti-inflammatory activities (30). Therefore, low level HDL-C is a remarkable risk factor for both CHD and CKD (30). Administration of Ros and Ator declined significantly AI levels in the present study. This finding is in agreement with the results of Munshi *et al.*, which underlines the efficacy of these drugs in improving hypercholesterolemia (1).

We found that feeding rats with HCD significantly increased serum OX-LDL levels. The raise in OX-LDL levels gives rise to the migration of macrophages and increased secretion of inflammatory cytokines and adhesion molecules in the vascular endothelium (31, 32). Endothelial dysfunction evoked by OX-LDL is an important factor in the development of atherosclerosis. Experimental evidence shows that OX-LDL declines nitric oxide (NO) synthesis in mesangial cells (8). Moreover, reduced levels of NO evoke synthesis of angiotensin II, increases expression of transforming growth factor beta (TGF-β) and inhibit plasminogen activator inhibitor-1 (PAI-1) (8). It also activates NADPH-oxidase that is the main source of ROS generation (10). OX-LDL decreases the activities of serum paraoxonase/arylesterase 1 (PON1) and as a result increases peroxidation of lipid profile (33). Collectively, these alterations lead to the acceleration of kidney disease and endothelial dysfunction as well as development of vascular sclerotic lesions (34). Kasiske *et al.* achieved similar results and showed the association between HCD and renal injury in their investigations (35). Likewise, Peric-Golia L and Peric-Golia M showed that HCD causes vascular damage and glomerulosclerosis in the kidneys (36).

According to our study, treatment with Ros and Ator reduces serum OX-LDL levels significantly (*P*<0.01). Apart from cholesterol reducing effects, statins possess pleotropic properties, inhibiting production of ROS by acting upon NADPH oxidase (8). Furthermore, statins are able to decrease biologic activities of angiotensin II and endothelin-1, improve renal function, and prevent atherosclerotic processes (30, 34). Additionally, statins increase NO synthesis and reduce hypertension related glomerular damages by inhibiting Rho expression (34, 37). Statins reduce the expression of leukocyte adhesion molecules and inhibit platelet aggregation (38-40). These agents also prevent lipid peroxidation by increasing PON1 activities (34). These findings suggest that Ros and Ator improve renal function in rats by the alleviation of oxidative stress and suppression of vascular inflammation/thrombosis, and attenuation of glomerular dysfunction. 

In order to evaluate lipid peroxidation, serum MDA levels were measured as a secondary by-product of oxidation. The aim of MDA measurement was to determine the rate of oxidative deterioration. Our findings showed that feeding HCD significantly increased serum MDA levels in HCD group compared to sham rats. Ros and Ator significantly reduced serum MDA levels compared with HCD group; Ros, however, was more effective than Ator in this respect. MDA itself is able to induce endothelial dysfunction and could give rise to the development of atherosclerosis. Munshi *et al.* indicated that feeding rats with high-fat-high-sucrose (HFHS) diet increases serum MDA levels and induces oxidative stress (1). In accordance with our findings, they also showed the capability of Ator in reducing serum MDA levels (1).

Serum urea and Cr levels are salient markers of kidney function. Serum urea and Cr levels had no significant difference between HCD and sham groups. In agreement with our findings, Adekunle *et al.* demonstrated that rats fed with atherogenic diet had no significant changes in their serum urea and Cr levels (41). Another study showed that serum urea levels in hyperlipidemic group were lower compared with control group. It may be due to less protein intake leading to less urea production (42).

Contrary to serum urea levels, treatment with Ros and Ator decreased serum Cr levels; however, this reduction was not statistically significant. Evidence show that treatment with HMGCOA reductase inhibitors decreases serum Cr levels and increases glomerular filtration rate in the kidneys (43).

Our findings revealed that HCD induces histopathological alterations such as deposition of numerous lipid droplets, especially in the proximal tubules, hemorrhage, nuclear pyknosis, karyolisis, infiltration of inflammatory cells, foam cell accumulation in the glomeruli, increased mesangial cell proliferation, vacuolated cytoplasm of proximal and distal tubules, tubular atrophy associated with tubular dilatation and loss of brush border in the tubular epithelium compared with control and sham groups. These findings are in agreement with previous studies (4, 10). Selim *et al.* showed that hyperlipidemia could damage glomerular and tubular cells in the kidneys (36). According to our study, administration of Ros and Ator significantly decreased HCD induced histopathological changes in the renal tissues of rats. The improvements in the Ator group were more pronounced than the Ros receiving animals. It seems that Ator, the lipophilic statin, easily enter into the tissues by passive diffusion as compared to Ros which is a hydrophilic statin; therefore, Ator could potentially be more effective in the treatment of glomerulosclerosis and improving renal function. Overall, treatment with Ros and Ator leads to alleviations in renal hypertrophy in hypercholesterolemic rats.

Rats were fed with HCD in 3 (HCD, HCD+Ros, and HCD+Ator) groups for 8 weeks. From the week 9, rats in groups IV (HCD+Ros) and V (HCD+Ator) were received Ros and Ator by intra-gastric gavage for 10 weeks

HCD, rats fed with hypercholesterolemic diet; HCD+Ros, rats fed with hypercholesterolemic diet+rosuvastatin (20mg/kg); HCD+Ator, rats fed with hypercholesterolemic diet+atorvastatin (20mg/kg)

**Table 1:** Effects of 5% cholesterol plus 0.1% cholic acid enriched diet on the serum lipid profile at week 8

**Animal group**	**Control group (I) (n=8)**		**HCD group (III) (n=8)**	
	**Before**	**After**	**Before**	**After**
**Cholesterol (mg/dl)**	49.37 ± 3.70	49.12 ± 6.66	50.12 ± 7.05	97.25 ± 33.93^a^
**Triglycerides (mg/dl)**	56.25 ± 6.80	52.37 ± 13.02	52.00 ± 27.42	50.25 ± 19.79
**HDL-C (mg/dl)**	30.03 ± 4.77	32.26 ± 5.80	31.20 ± 5.69	23.45 ± 4.47^a^
**LDL-C (mg/dl)**	8.08 ± 1.62	6.38 ± 2.32	8.02 ± 3.22	63.75 ± 31.99^a^
**VLDL-C (mg/dl)**	11.27± 1.40	10.47 ± 2.60	10.40 ± 5.48	10.05 ± 3.96

Control, rats fed with normal diet; HCD, rats fed with hypercholesterolemic diet. Values are expressed as the means±SD

LDL-C, low density lipoprotein cholesterol; VLDL-C, very low density lipoprotein cholesterol; HDL-C, high density lipoprotein cholesterol.

^a^ *P<*0.01 vs. Control

**Table 2:** Effects of rosuvastatin (Ros) and atorvastatin (Ator) on body weight and lipid profile of rats at week 10

**Animal group**	**Sham (II)** **(n = 8)**		**HCD group (III)** **(n = 8)**		**HCD + Ros (IV) ** **(n = 8)**		**HCD + Ator (V)** **(n = 8)**	
	**Before**	**After**	**Before**	**After**	**Before**	**After**	**Before**	After
**Weight (g)**	251.62 ± 15.24	264.37 ± 18.01	284.50 ± 27.08^a^	314.38 ± 30.52	283.25 ± 13.22^a^	292.00 ± 12.44	290.00 ± 12.81^a^	294.38 ± 8.10
**Cholesterol** **(mg/dl)**	50.1 ± 4.97	52.37 ± 9.87	97.25 ± 33.93^a^	91.87 ± 19.96	98.50 ± 29.15^a^	46.00 ± 5.95^c^	94.50 ± 28.47^a^	54.25 ± 8.32^c^
**Triglycerides** **(mg/dl)**	58.25± 10.02	47.87 ± 26.01	50.25 ± 19.79	57.62 ± 11.34	50.75 ± 5.28	25.50 ± 5.07^c^	48.00 ± 11.10	27.00 ± 7.54^c^
**HDL-C (mg/dl)**	32.04 ± 3.43	34.47 ± 6.51	23.45 ± 4.47^a^	23.37 ± 6.75	22.97 ± 4.72^a^	34.25 ± 5.86^b^	24.20 ± 5.12^a^	33.00 ± 4.40^b^
**LDL-C (mg/dl)**	6.40 ± 3.33	8.32 ± 4.20	63.75 ± 31.99^a^	56.97 ± 16.34	65.37 ± 26.06^a^	6.67 ± 1.99^c,d^	60.67 ± 25.87^a^	18.35 ± 3.58^c^
**VLDL-C (mg/dl)**	11.65 ± 2.00	9.57 ± 5.20	10.05 ± 3.96	11.52 ± 2.27	10.15 ± 1.06	5.10 ± 1.01^c^	9.60 ± 2.22	5.40 ± 1.50^c^
**AI**	0.57 ± 0.14	0.53 ± 0.22	3.08 ± 0.83^a^	3.05 ± 0.87	3.53± 0.93^a^	0.37± 0.086^c,d^	2.89 ± 0.69^a^	0.73 ± 0.18^c^

Sham, rats fed with normal diet+water gavage; HCD, rats fed with hypercholesterolemic diet; HCD+Ros, rats fed with hypercholesterolemic diet+Ros (20 mg/kg); HCD+Ator, rats fed with hypercholesterolemic diet+Ator (20 mg/kg). Values are expressed as the means±SD

Ator, atorvastatin; Ros, rosuvastatin; LDL-C, low density lipoprotein cholesterol; VLDL-C, very low density lipoprotein cholesterol; HDL-C, high density lipoprotein cholesterol; AI, Atherogenic index

^a^ *P<*0.01 vs. sham; ^b^ *P<*0.05 vs. HCD; ^c^ *P<*0.01 vs. HCD; ^d^ *P<*0.01 vs. HCD+Ator

**Table 3:** Effect of rosuvastatin (Ros) and atorvastatin (Ator) on the biochemical parameters of rats at week 10

**Animal group**	**Sham (II)** **(n=8)**		**HCD (III)** **(n=8)**		**HCD + ROS (IV)** ** (n=8)**		**HCD + ATOR (V)** ** (n=8)**	
	**Before**	**After**	**Before**	**After**	**Before**	**After**	**Before**	**After**
**Urea (mg/dl)**	50.37 ± 7.00	53.00 ± 6.76	44.00 ± 8.71	47.75 ± 6.54	45.87 ± 7.88	50.87 ± 6.55	45.50 ± 10.65	52.37 ± 6.58
**Creatinine (mg/dl)**	0.44 ± 0.10	0.41 ± 0.03	0.47 ± 0.13	0.45 ± 0.10	0.46 ± 0.04	0.34 ± 0.07	0.47 ± 0.22	0.37 ± 0.06
**Calcium (mg/dl)**	8.17 ± 0.92	8.56 ± 0.96	8.9 ± 0.74	8.97 ± 0.89	9.17 ± .94	8.18 ± 0.61	8.59 ± 0.45	8.40 ± 0.71
**Phosphorus (mg/dl)**	5.07 ± 0.85	5.45 ± 1.08	5.45 ± 0.96	5.32 ± 1.00	5.38 ± 0.58	5.33 ± 0.07	5.40 ± 0.74	5.32 ± 1.01

Sham, rats fed with normal diet+water gavage; HCD, rats fed with hypercholesterolemic diet; HCD+Ros, rats fed with hypercholesterolemic diet+Ros (20 mg/kg); HCD+Ator, rats fed with hypercholesterolemic diet+Ator (20 mg/kg). Values are expressed as the means±SD. No differences between groups before and after treatment

**Table 4:** Effect of rosuvastatin (Ros) and atorvastatin (Ator) on the serum markers of oxidative stress in rats

Animal group	Sham (II) (n=8)		HCD (III) (n=8)		HCD + ROS (IV) (n=8)		HCD + ATOR (V) (n=8)	
	Before	After	Before	After	Before	After	Before	After
MDA (ng/ml)	2.20 ± 0.20	2.26 ± 0.16	5.80 ± 0.26^a^	5.90 ± 0.15	5.79 ± 0.32^a^	3.35 ± .24^b,c^	5.77 ± 0.21^a^	3.91 ± 0.20^b^
OX-LDL (µg/ml)	1.95 ± 0.09	1.97 ± 0.13	2.32 ± 0.16^a^	2.35 ± 0.26	2.31 ± 0.06^a^	1.66 ± 0.07^b,c^	2.30 ±0.11^a^	1.94 ± 0.18^b^

Sham, rats fed with normal diet+water gavage; HCD, rats fed with hypercholesterolemic diet; HCD+Ros, rats fed with hypercholesterolemic diet+Ros (20 mg/kg); HCD+Ator, rats fed with hypercholesterolemic diet+Ator (20 mg/kg). Values are expressed as the means±SD

MDA, malondialdehyde; OX-LDL, oxidized low density lipoprotein

^a^ *P<*0.01 vs. sham; ^b^ *P<*0.01 vs. HCD; ^c^ *P<* 0.01 vs. HCD+Ator

**Table 5.** Effect of rosuvastatin (Ros) and atorvastatin (Ator) on tubulointerstitial damage in kidney tissues of rats

**HCD + ATRO (V)** **(n=8)**	**HCD + ROS(IV)** **(n=8)**	**HCD (III)** **(n=8)**	**Sham (II)** **(n=8)**	**Control ** **)** **I** **(** **(n=8)**	**Variable**
1.08 ± 0.09^b,c^	1.46 ± 0.19^b^	2.00 ± 0.13 ^a^	0.15 ±0.07	0.12 ± 0.08	**TID**

Control, rats fed with normal diet; Sham, rats fed with normal diet+water Gavage; HCD, rats fed with hypercholesterolemic diet; HCD+ROS, rats fed with hypercholesterolemic diet+Ros (20 mg/kg); HCD+Ator, rats fed with hypercholesterolemic diet+Ator (20 mg/kg); TID, tubulointerstitial damage. Values are expressed as the means±SD

Semi quantitative analysis of TID scores in the study groups. The TID score in HCD group was significantly (*P=*0.001) higher than the control and sham groups. The TID scores in (Ator+HCD) and (Ros+HCD) were semi quantitative lower than HCD group

^a^ *P<*0.01vs. Sham and control group; ^b^ *P<*0.01 vs. HCD; ^c^ *P<*0.01vs. HCD+Ros

Control, rats fed with normal diet; Sham, rats fed with normal diet + water Gavage; HCD, rats fed with hypercholesterolemic diet; HCD+ROS, rats fed with hypercholesterolemic diet+Ros (20 mg/kg); HCD+Ator, rats fed with hypercholesterolemic diet+Ator (20 mg/kg)

Foam cells (→), tubular atrophy (→), lipid droplets (→) tubular dilatation (→)

## Conclusion

This study shows that cholesterol rich diet in rats induces hypercholesterolemia and increases oxidative stress. Hypercholesterolemia accelerates the development of renal histological alterations. These findings underline that apart from cholesterol lowering effects, Ros and Ator could potentially be protective against renal damages; moreover, the lipophilic Ator is superior to hydrophilic Ros in ameliorating renal injury parameters in the HCD induced hypercholesterolemic rats.

## References

[1] Munshi RP, Joshi SG, Rane BN (2014). Development of an experimental diet model in rats to study hyperlipidemia and insulin resistance, markers for coronary heart disease. Indian J Pharmacol.

[2] Csonka C, Sárközy M, Pipicz M, Dux L, Csont T (2016). Modulation of hypercholesterolemia-induced oxidative/nitrative stress in the heart. Oxid Med Cell Longev.

[3] Kose E, An T, Kikkawa A, Matsumoto Y, Hayashi H (2014). Effects on serum uric acid by difference of the renal protective effects with atorvastatin and rosuvastatin in chronic kidney disease patients. Biol. Pharm. Bull.

[4] Belguithhadriche O, Bouaziz M, Jamoussi K, Elfeki A, Makniayedi F (2014). Renoprotective effects of fenugreek seeds against oxidative stress in hypercholesterolemic fed Rats. J Med Food.

[5] Sundaram M, Palaneeswari S, Nagarajan S, Devi M, Jagdeeshwaran A (2014). Chronic kidney disease - effect of oxidative stress. Chin J Biol.

[6] Jaqueto M, Delfino VDA, Bortolasci CC, Barbosa DS, Morimoto HK, Frange RFN (2016). Are PTH levels related to oxidative stress and inflammation in chronic kidney disease patients on hemodialysis?. J Bras Nefrol.

[7] Elhemely MA, Omar HA, Ain-Shoka AA, El-Latif HAA, Abo-youssef AM, El Sherbiny GA (2014). Rosuvastatin and ellagic acid protect against isoproterenol-induced myocardial infarction in hyperlipidemic rats. BJBAS.

[8] Lee HS, Song CY (2009). Oxidized low-density lipoprotein and oxidative stress in the development of glomerulosclerosis. Am J Nephrol.

[9] Lovrić J, Mesić M, Macan M, Koprivanac M, Kelava M, Bradamante V (2008). Measurement of malondialdehyde (MDA) level in rat plasma after simvastatin treatment using two different analytical methods. Period Biol.

[10] Sastre C, Rubio-Navarro A, Buendía I, Gómez-Guerrero C, Blanco J, Mas S (2013). Hyperlipidemia-associated renal damage decreases Klotho expression in kidneys from ApoE knockout mice. PLoS One.

[11] Anagnostis P, Adamidou F, Slavakis A, Polyzos SA, Selalmatzidou D, Panagiotou A (2014). Comparative effect of atorvastatin and rosuvastatin on 25-hydroxy-vitamin D levels in non-diabetic patients with dyslipidaemia: a prospective randomized open-label pilot study. Open Cardiovasc Med J.

[12] Girardi JM, Farias RE, Ferreira AP, Raposo NRB (2011). Rosuvastatin prevents proteinuria and renal inflammation in nitric oxide-deficient rats. Clinics.

[13] Schachter M (2005). Chemical, pharmacokinetic and pharmacodynamic properties of statins: an update. Fundam Clin Pharmacol.

[14] Arshad AR (2014). Comparison of low-dose rosuvastatin with atorvastatin in lipid-lowering efficacy and safety in a high-risk pakistani cohort: an open-label randomized trial. J Lipids.

[15] Kim MC, Ahn Y, Jang SY, Cho KH, Hwang SH, Lee MG (2011). Comparison of clinical outcomes of hydrophilic and lipophilic statins in patients with acute myocardial infarction. Korean J Intern Med.

[16] Večeřa R, Zachařová A, Šiller M, Matušková Z, Škottová N, Anzenbacherová E (2012). The influence of rosuvastatin on liver microsomal CYP2C6 in hereditary hypertriglyceridemic rat. Neuro Endocrinol Lett.

[17] Nurkalem Z, Yildirimtürk Ö, Özcan KS, Kul Ş, Çanga Y, Satılmış S (2014). The effect of rosuvastatin and atorvastatin on erectile dysfunction in hypercholesterolaemic patients. Kardiol Pol.

[18] Fellström B, Zannad F, Schmieder R, Holdaas H, Jardine A, Rose H (2005). Effect of rosuvastatin on outcomes in chronic haemodialysis patients–design and rationale of the AURORA study. Curr Control Trials Cardiovasc Med.

[19] Chan C (2005). Hyperlipidaemia in chronic kidney disease. Ann. Acad. Med. Singap..

[20] Park J-S, Kim Y-J, Choi J-Y, Kim Y-N, Hong T-J, Kim D-S (2010). Comparative study of low doses of rosuvastatin and atorvastatin on lipid and glycemic control in patients with metabolic syndrome and hypercholesterolemia. Korean J Intern Med.

[21] Jones PH, Davidson MH, Stein EA, Bays HE, McKenney JM, Miller E (2003). Comparison of the efficacy and safety of rosuvastatin versus atorvastatin, simvastatin, and pravastatin across doses (STELLAR Trial). Am J Cardiol.

[22] Schulpis K, Karikas GA (1998). Serum cholesterol and triglyceride distribution in 7767 school-aged Greek children. Pediatrics.

[23] Lapenna D, Ciofani G, Pierdomenico SD, Giamberardino MA, Cuccurullo F (2001). Reaction conditions affecting the relationship between thiobarbituric acid reactivity and lipid peroxidesin human plasma. Free Radic Biol Med.

[24] Reisin E, Liao J, Lee BS, Larroque M, Aguilar EA, Morse SA (2009). Effect of the HMG-CoA reductase inhibitor rosuvastatin on early chronic kidney injury in obese zucker rats fed with an atherogenic diet. Am J Med Sci.

[25] Abrass CK (2004). Cellular lipid metabolism and the role of lipids in progressive renal disease. Am J Nephrol.

[26] van Himbergen TM, Matthan NR, Resteghini NA, Otokozawa S, Ai M, Stein EA (2009). Comparison of the effects of maximal dose atorvastatin and rosuvastatin therapy on cholesterol synthesis and absorption markers. J Lipid Res.

[27] Crevar-Sakač M, Vujić Z, Kotur-Stevuljević J, Ivanišević J, Jelić-Ivanović Z, Milenković M (2016). Effects of atorvastatin and artichoke leaf tincture on oxidative stress in hypercholesterolemic rats. Vojnosanit Pregl.

[28] Agarwal R (2007). Effects of statins on renal function. Mayo Clin. Proc.

[29] Xilifu D, Abudula A, Rehemu N, Zhao L, Zhou X, Zhang X (2014). Effect of rosuvastatin on hyperuricemic rats and the protective effect on endothelial dysfunction. Exp Ther Med.

[30] Kilit C, Koçak FE, Kilit TP (2017). Comparison of the effects of high-dose atorvastatin and high-dose rosuvastatin on oxidative stress in patients with acute myocardial infarction: A pilot study. Turk Kardiyol Dern Ars.

[31] Nishikido T, Oyama J-i, Keida T, Ohira H, Node K (2016). High-dose statin therapy with rosuvastatin reduces small dense LDL and MDA-LDL: the standard versus high-dose therApy with Rosuvastatin for lipiD lowering (SARD) trial. J Cardiol.

[32] Shehata AM, Yousef OM (2010). Physiological studies on the risk factors responsible for atherosclerosis in rats. Nat and Sci.

[33] Samani KG, Farrokhi E (2014). Effects of cumin extract on oxLDL, paraoxanase 1 activity, FBS, total cholesterol, triglycerides, HDL-C, LDL-C, Apo A1, and Apo B in the patients with hypercholesterolemia. Int J Health Sci.

[34] Beltowski J (2005). Statins and modulation of oxidative stress. Toxicol. Mech. Methods.

[35] Kasiske BL, O’Donnell MP, Schmitz PG, Kim Y, Keane WF, Daniels F (1990). Renal injury of diet-induced hypercholesterolemia in rats. Kidney Int..

[36] Selim ME, Yousef OM, Hamid SH, Aleisa NA (2013). Hyperlipidemia aggravated renal disease in bacteremic male albino rats. J Med Sci.

[37] Deng J, Wu G, Yang C, Li Y, Jing Q, Han Y (2015). Rosuvastatin attenuates contrast-induced nephropathy through modulation of nitric oxide, inflammatory responses, oxidative stress and apoptosis in diabetic male rats. J Transl Med.

[38] Li D, Chen H, Romeo F, Sawamura T, Saldeen T, Mehta JL (2002). Statins modulate oxidized low-density lipoprotein-mediated adhesion molecule expression in human coronary artery endothelial cells: role of LOX-1. J Pharmacol Exp Ther.

[39] Yilmaz MI, Baykal Y, Kilic M, Sonmez A, Bulucu F, Aydin A (2004). Effects of statins on oxidative stress. Biol Trace Elem Res.

[40] Panonnummal R, Yarkey J, Dinoop D (2013). Are statins nephroprotective?: a dose dependent study in albino rats. Int J Pharm Pharm Sci.

[41] Adekunle A, Adedeji A, Oyewo E, Adedosu O, Omotoso A (2013). Hyperlipidemia induced by atherogenic diet enhanced oxidative stress in the kidney and inflammatory responses: an in vivo study. Asian J Nat Appl Sci.

[42] Matos SL, Paula Hd, Pedrosa ML, Santos RCd, Oliveira ELd, Chianca Júnior DA (2005). Dietary models for inducing hypercholesterolemia in rats. Braz Arch Biol Techn.

[43] Srinivas M, Annapurna A, Reddy YN (2008). Anti-atherosclerotic effect of atorvastatin and clopidogrel alone and in combination in rats. Indian J Exp Biol.

